# Broken beyond repair: TA system ParE toxins mediate effective gyrase inhibition without driving resistance

**DOI:** 10.1128/jb.00416-24

**Published:** 2025-03-03

**Authors:** Chih-Han Tu, Shengfeng Ruan, Michelle Holt, Christina R. Bourne

**Affiliations:** 1 Department of Chemistry and Biochemistry, University of Oklahoma 522128 , Norman, Oklahoma, USA; NCBI, NLM, National Institutes of Health, Bethesda, Maryland, USA

**Keywords:** toxin antitoxin, gyrase inhibition, mutation frequency, ParE toxin, bacterial cell viability, antimicrobial susceptibility

## Abstract

DNA gyrase is an essential bacterial-specific type IIA topoisomerase that corrects DNA overwinding during transcription and replication. Compounds capable of stabilizing gyrase-mediated double-strand DNA breaks are valuable antibacterials; however, these can trigger error-prone repair, potentially inducing DNA mutations leading to antimicrobial resistance. ParE toxin proteins, which belong to a family of type II toxin-antitoxin systems, inhibit DNA gyrase and promote the persistence of double-strand DNA breaks. However, it is unclear if the ParE-induced gyrase inhibition is equivalent for all ParE family members, or if any mutations arise and can accumulate to cause antibiotic resistance. Selected chromosomal ParE toxins were examined for toxicity to their native bacterial hosts, and the frequency of mutations and impact on susceptibility to selected antibiotics were assessed. Our results show that ParE toxins from *Burkholderia cenocepacia*, *Mycobacterium tuberculosis*, *Pseudomonas aeruginosa*, and *Vibrio cholerae* exert potent toxicities toward the native cells, whereas one tested ParE toxin from *P. aeruginosa* was not toxic. The contribution to toxicity of the ParE toxin C-terminal amino acid sequences was examined using two lab-generated chimeric ParE toxins; our results demonstrate that this region did not impact the toxicity level. Our study finds that the relative potency of individual ParE toxins correlates with increases in mutation frequency. While some ParE toxins induced limited collateral sensitivity to selected antibiotics, no increases in MIC values were found. Overall, this study demonstrates the relative toxicity of different ParE toxins. Importantly, the toxicity appears to result in loss of viability before productive resistance-inducing mutations can accumulate.

**IMPORTANCE:**

Toxin-antitoxin (TA) systems can halt growth or kill cells when the toxin protein engages with the host cell target. In the ParDE TA system, the toxin ParE inhibits DNA gyrase, resulting in loss of viability that phenocopies fluoroquinolone antibiotics. Our study demonstrates that ParE toxins increase the frequency of mutations, presumably by a mechanism similar to fluoroquinolone antibiotics. These increases scale to the resulting toxicity, and importantly, these mutations do not accumulate into productive antibacterial resistance. This suggests that ParE toxins are not intrinsic drivers of resistance and, if the molecular mechanism can be harnessed, could generate a new class of gyrase inhibitors.

## INTRODUCTION

Type II toxin-antitoxin (TA) systems are composed of bacterial toxins and co-evolved specifically paired antitoxin proteins that neutralize, and thus protect, the host bacterial cell (1–3). TA systems are pervasive in the prokaryotic world, with just over 4,000 predicted ParDE-subfamily type II TA systems (4). ParDE TA systems are carried on mobile genetic elements, such as plasmids, where they enforce retention by “addicting” bacterial cells to the co-encoded labile cognate antitoxin to maintain continual toxin neutralization (5). Plasmid-born *parDE* loci are enriched on IncI and IncF type plasmids that impart antibacterial resistance to *Enterobacteriaceae* and *Salmonella* species (6), as well as a *Yersinia*-specific plasmid carrying a virulence-mediating type III secretion system (7). ParDE TA systems have also been captured into bacterial genomes, whereby a similar selective strategy retains so-called “accessory” chromosomes, such as *parDE* operons located in pathogenicity islands in *Vibrio cholerae* (Vc) (8). Other chromosomal ParDE TA systems are embedded within prophage islands where they impact phage activation, such as the Pf4-type phage that directly promotes biofilm formation by *Pseudomonas aeruginosa* (Pa) (9). Alternative roles for ParDE systems include a chromosomal version in *Caulobacter crescentus* (Cc) that is essential to trigger the release of extracellular DNA needed to seed biofilm formation (10). We and others have speculated that components of chromosomal TA systems may cross-react with plasmid-derived TA systems in an ongoing adaptation between bacteria and invading genetic materials (11, 12).

Our long-standing interests are in the potential usefulness of the ParDE-type subfamily by blocking ParD antitoxin interaction with its cognate ParE toxin, thereby promoting inhibition of the essential bacterial topoisomerase DNA gyrase (13). Inhibition of DNA gyrase by ParE toxins mimics that of commonly used fluoroquinolone antibiotics, whereby the catalytic cycle is blocked midway and results in the accumulation of double-stranded DNA breaks (14–16). Expression of multiple ParE toxins (17–19) and other gyrase-inhibiting proteins (reviewed in ref. [14]) has been observed to induce a filamentous phenotype, consistent with DNA damage resulting from inhibition of DNA gyrase. Fragmentation of genomic DNA triggers damage response pathways, including the SOS response that includes error-prone DNA polymerases (e.g., II, IV, and V) (20, 21). These are capable of replicating across damaged DNA strands and bypassing DNA lesions, although this increases the likelihood of mutagenesis (22, 23). This response has been documented to increase the frequency of mutations upon administration of commonly used gyrase inhibitors, especially the fluoroquinolone class of antibiotics (24–26). In the current work, we questioned if ParE toxins would have a similar impact and assessed the frequency of mutations as a function of ParE induction using a modified fluctuation assay (27, 28).

Our motivation for studies of ParDE systems lies in the potential to manipulate their pairings and, in so doing, potentially allow excess free ParE toxin to exert impacts by inhibition of the validated antibacterial target, DNA gyrase, including in ciprofloxacin (CIP)-resistant strains (18, 29). However, this approach requires careful consideration of potential downstream effects, including mutagenic drivers of antibacterial resistance, as well as relative toxicities of different ParE family members. Many published accounts, including ours, rely on an over-expression system to assess the impacts of ParE toxins (15, 16, 19, 30). We previously characterized differences in toxicity of ParE proteins when expressed in an *E. coli* host commonly employed for recombinant protein production (17). There are clear drawbacks to the recombinant expression approach, including issues with protein folding, non-native expression levels, formation of inclusion bodies (31, 32), and reliance on maintaining stable plasmid copy numbers (33). Native expression remains infeasible, however, due to lack of knowledge on native TA promoter activation. We reasoned that impacts arising from ParE inhibition are also heavily dependent on native host pathways and that protein folding should be more favorable in the native context.

In the current study, we screened bacterial cell viability as a function of induction concentrations for ParE toxins from *Burkholderia cenocepacia* (Bc), *Mycobacterium tuberculosis* (Mt), *P. aeruginosa* (Pa), and *V. cholerae* (Vc), in native hosts and/or in *E. coli* strain MG1655, a common surrogate host in TA system studies (33, 34). Cultures were screened for the frequency of mutations upon ParE induction, and we found a general correlation between toxicity and increased mutation frequencies. Increases in mutation frequencies did not correlate with decreased antibiotic susceptibility and, instead, reveal some collateral sensitivity imparted by ParE-mediated toxicity. Overall, these studies demonstrate a spectrum of toxicity levels mediated by different ParE toxins, the origin of which appears to lie within each ParE sequence but, as we establish, is independent of the varied sequence found in the unstructured C-terminal end of ParE toxins. The outcomes of these assays support the potential use of ParE toxins or mimics thereof in an antibiotic-like approach and highlight that resulting mutagenic effects are not expected to promote the development of antibacterial resistance.

## RESULTS

ParE toxins were identified from annotations in the toxin-antitoxin database (TADB) (4), and seven chromosomal ParDE systems were selected for this study (Table S1). The identified sequences were amplified and subsequently cloned into appropriate vectors with expression driven by inducible promoters (Table 3).

### Induction of ParE toxins decreases viability concomitant with increased mutagenic frequency

To measure the impact of ParE toxin expression during growth in liquid cultures, a range of induction strengths was used, and resulting viable cell counts were quantified as colony-forming units per milliliter (CFUs/mL). Furthermore, the mutation frequency following ParE toxin induction was measured using an adapted “fluctuation assay.” This relies on selection for single point mutations that give rise to rifampicin (RIF) or trimethoprim (TMP) resistance when plated on an excess of the experimentally determined minimum inhibitory concentration (MIC) values (Table 1) (28).

**TABLE 1:** MICs in μg/mL for RIF, TMP, and CIP against the bacterial strains in test media used in the current studies

Bacterial strain	RIF	Published value	TMP	Published value	CIP	Published value
*B. cenocepacia* J2315	32–64	32^*a*^ (35)	n*.*t.	–	4	4^*b*^ (36)
*P. aeruginosa* PA14^*c*^	>500	u*.*r.	100	u*.*r.	0.05	0.064^*b*^ (37)
*P. aeruginosa* PAO1^*c*^	n*.*t.	u*.*r.	100	>32^*d*^ (38)	0.07	0.063–0.09^*b*^ (39, 40)
*V. cholerae* N16961^*c*^	75	1.0–1.2 (24, 41)	n*.*t.	–	0.07	0.079–0.5 (24, 41)
*E. coli* MG1655^*b*^	10	12–25 (42, 43)	0.25	0.2–1 (44, 45)	0.04	0.016–0.035 (46, 47)

^*a*^ MIC tested in cation-adjusted Muller Hinton broth. n*.*t., not tested; u*.*r., extensive literature searches did not reveal a published value.

^*b*^ MIC tested in LB medium.

^*c*^ MIC tested in M9 minimal medium.

^*d*^ MIC tested in tryptic soy broth.

**Fig 1:**
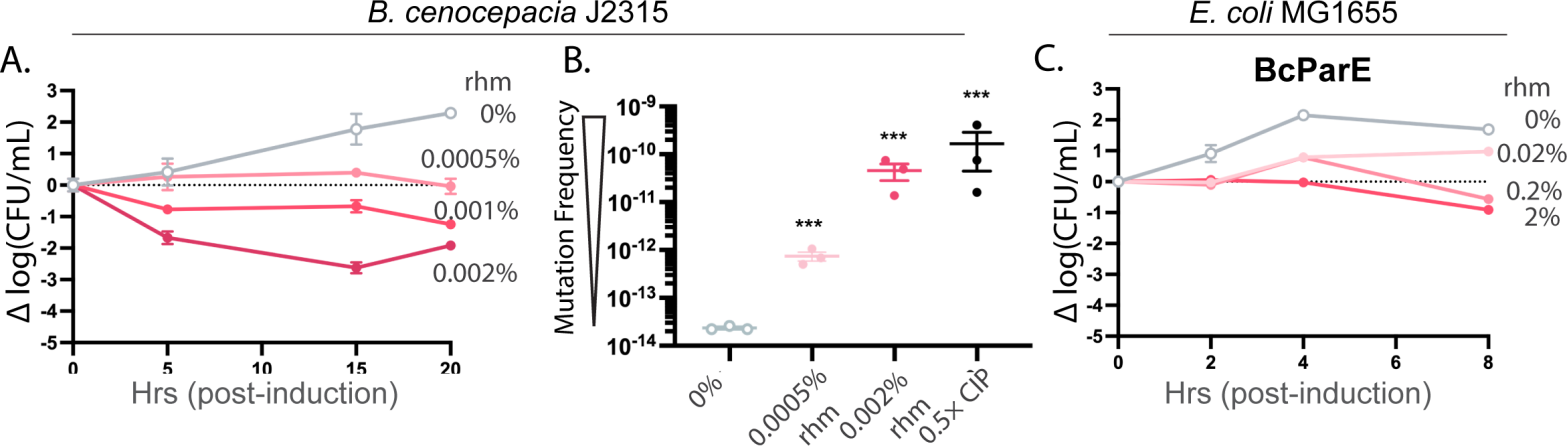
The BcParE toxin induces loss of viability that correlates with a dose-dependent increase in mutagenic frequency. (**A**) Induction of BcParE encoded in the rhamnose (rhm)-inducible pSCrha vector harbored in its native host *B. cenocepacia* J2315 causes stagnation and loss of viability more than 4-log values at timepoints equivalent to approx. 5 cell doublings (15 h timepoint with a 3 h doubling time). (**B**) Increasing stagnation or toxicity at the 15 h timepoint triggers increases in mutation frequency, with the highest impacts comparable to treatment with gyrase inhibitor ciprofloxacin (CIP). (**C**) In contrast, the BcParE toxin has modest impacts on the viability of *E. coli* MG1655. Viability data are presented as change from initial CFU/mL (dashed line); all data are displayed as standard error of the mean (SEM) calculated from three independent experiments. Mutation frequency of ParE-induced or CIP-treated cells was compared to that of the 0% induction cells using an unpaired two-tailed Student’s *t*-test: ***P < 0.001.

The doubling time of *B. cenocepacia* is significantly slower than the other tested bacteria (approx. 3 h versus 20–80 min, Table S2) and is relatively insensitive to induction with arabinose, requiring different timing and inducible promoter (rhamnose, rhm) for these experiments (48, 49). A potent reduction in cell viability is evident, with the lowest inducer concentration initially displaying stagnation in growth while others have immediate losses (Fig. 1A). These samples were captured at the 15 h timepoint, and the frequency of mutations was measured. Stagnated growth correlated with an approx. 2-log increase in mutation frequency (Fig. 1B). Viable cell counts follow the trend of higher induction triggering a greater loss of viable cells relative to the starting count, with the largest impact occurring at the 15 h timepoint and resulting in almost 99.99% loss (4-log), driving the frequency of mutations to levels comparable to the positive control CIP, which is an approx. 4-log increase from the uninduced control (Fig. 1B). In contrast, the BcParE toxin has apparent mild toxicity in *E. coli* MG1655, a commonly used surrogate strain for TA system studies, with an overall suppression of growth that sustained cell viability at starting levels with essentially no appreciable cell death (Fig. 1C). No impact on growth was observed for control samples (vector with no insert, grown and induced in parallel, Fig. S1), highlighting the specific impact of BcParE.

**Fig 2:**
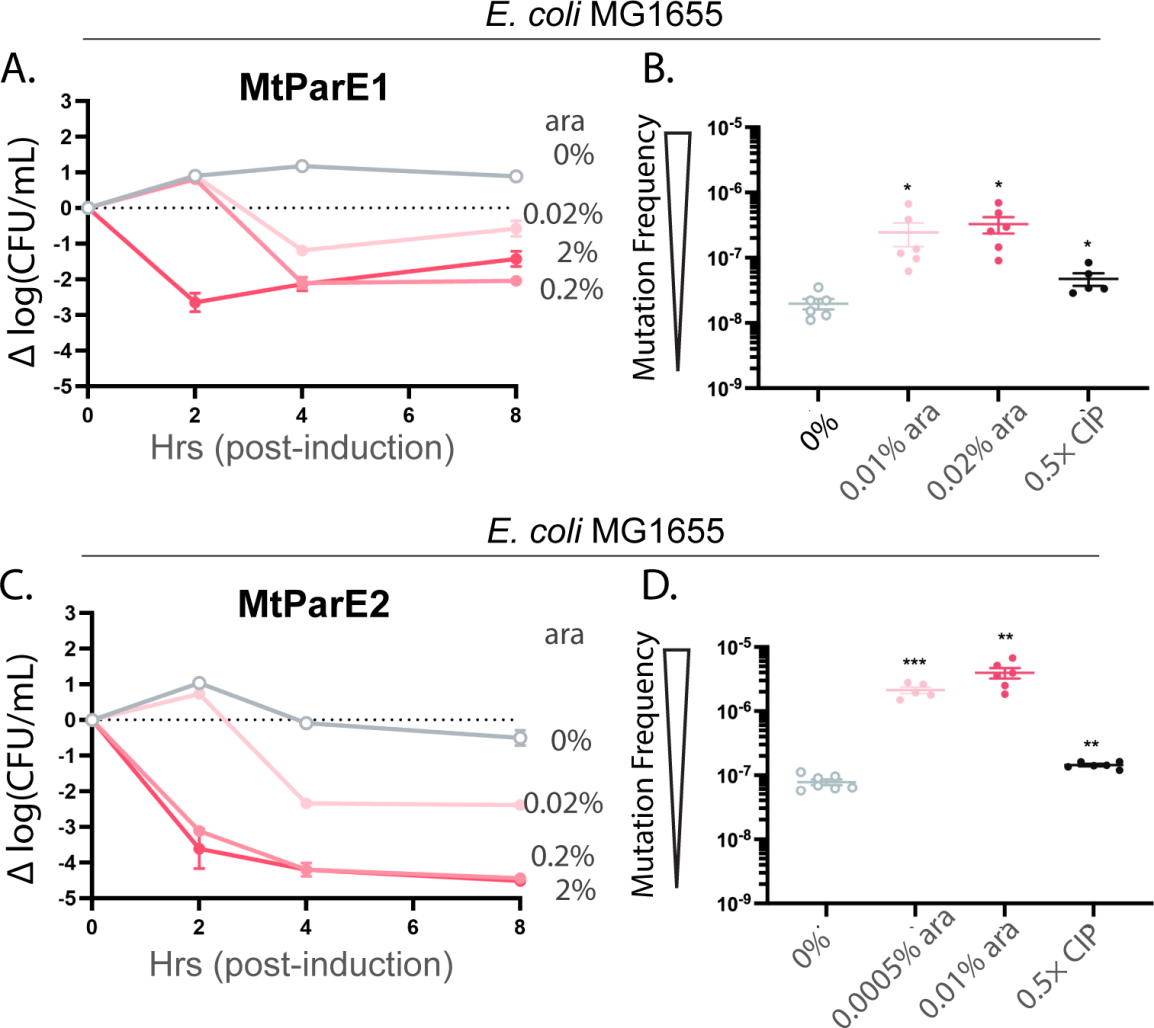
The two ParE toxins encoded in the genome of *M. tuberculosis* induce loss of viability to surrogate bacterial host *E. coli* MG1655 that correlates with a dose-dependent increase in mutagenic frequency. (**A**) Induction of MtParE1 and (**C**) MtParE2 from the arabinose (ara)-inducible pMindBAD vector causes a loss of viability to *E. coli* with a stronger effect noted for MtParE2. (**B**) Mutation frequency is increased for both MtParE1 and (**D**) MtParE2, with even mild induction producing frequencies in excess of the positive control gyrase-inhibiting ciprofloxacin (CIP) treatment. Viability data are presented as change from initial CFU/mL (dashed line); all data are displayed as standard error of the mean (SEM) calculated from three independent experiments. Mutation frequency of ParE-induced or CIP-treated cells was compared to that of the 0% induction cells using an unpaired two-tailed Student’s *t*-test: *P < 0.05, **P < 0.01, and ***P < 0.001.

Two ParE toxins encoded in the genome of *M. tuberculosis* were tested for toxicity against a surrogate strain rather than the native host strain due to biosafety limitations. These were cloned into a modified pMind vector with the tetRO promoter replaced by an arabinose-inducible (araBAD) promoter to assist with consistency between constructs (Table 3). Both MtParE1 and MtParE2 toxins exhibited a dose-dependent toxicity profile in *E. coli* MG1655 cells (Fig. 2A and C). The MtParE2 toxin exhibited stronger toxicity than the MtParE1 toxin, as evidenced by lower cell counts at the same induction levels. Specifically, MtParE1 toxin expression led to a maximum of approx. 2.7-log reduction in cell counts at a 2% induction level within 2 h of induction (Fig. 2A), whereas MtParE2 toxin expression led to an approx. 4.5-log reduction after 8 h of induction at both 0.2% and 2% induction levels (Fig. 2C). These levels of toxicity correlated to dose-dependent increases in mutation frequency even at substantially lowered induction strengths (Fig. 2B and D). Specifically, MtParE1 toxins led to an approx. 1-log increase in mutation frequency at a 0.02% induction level (Fig. 2B), while MtParE2 toxin led to a 2.3-log increase in mutation frequency at a 0.001% induction level (Fig. 2D). Interestingly, both MtParE1 and MtParE2 toxins led to higher increases in mutation frequency than the CIP positive control.

**Fig 3:**
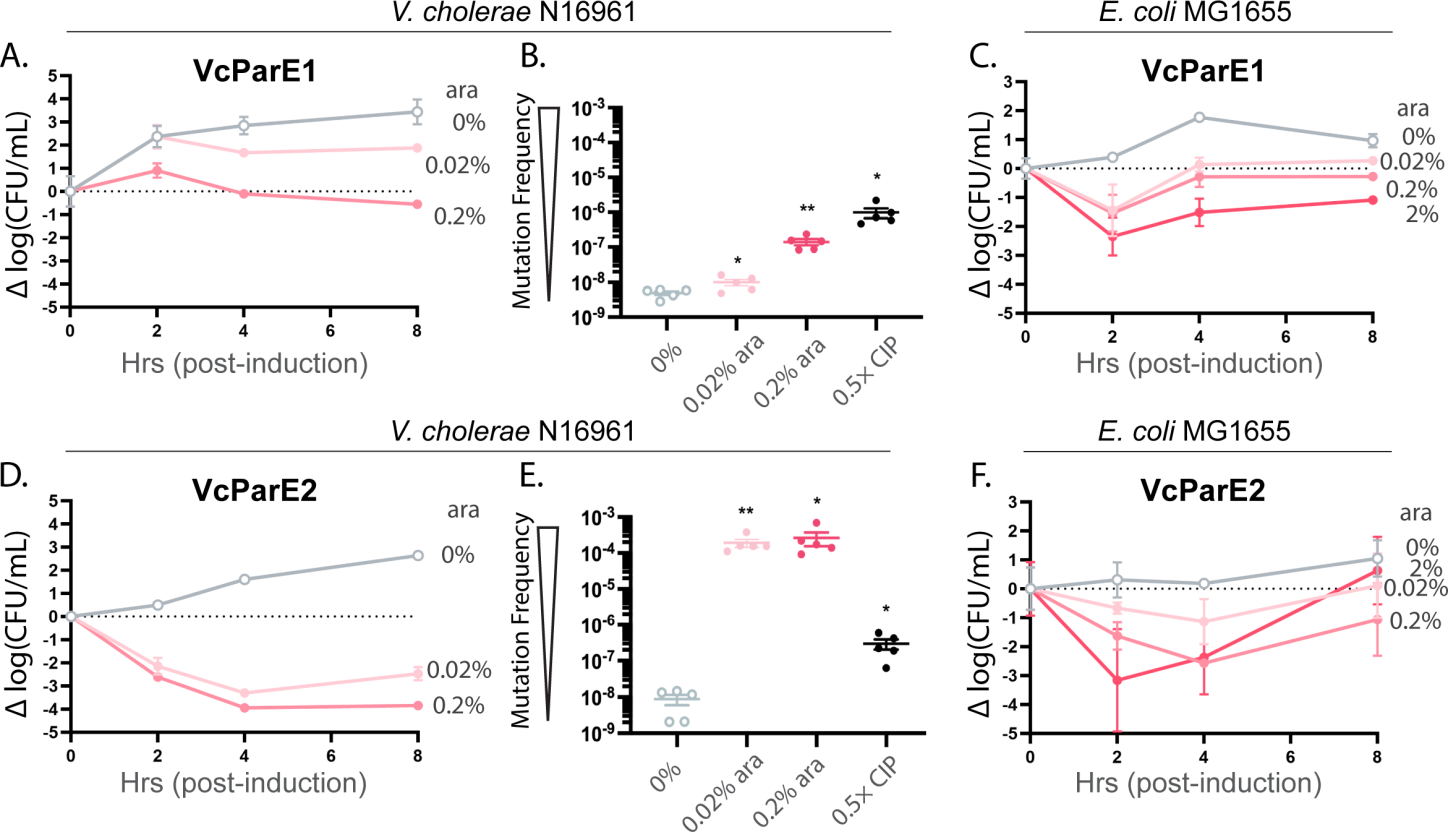
The two ParE toxins encoded in the genome of *V. cholerae* exert toxicity and trigger corresponding increases in mutation frequencies. (**A**) Induction of VcParE1 and (**D**) VcParE2 cause dose-dependent loss of viability with a stronger effect mediated by VcParE2. The level of toxicity correlates directly with the increase in mutation frequency (**B, E**), with a much stronger effect seen for VcParE2 at more than a 4-log increase. Both toxins also display toxicity in *E. coli* MG1655 (**C, F**) although to a lower level than to the native host. Viability data are presented as change from initial CFU/mL (dashed line); all data are displayed as standard error of the mean (SEM) calculated from three independent experiments. Mutation frequency of ParE-induced or CIP-treated cells was compared to that of the 0% induction cells using an unpaired two-tailed Student’s *t*-test: *P < 0.05 and **P < 0.01.

*V. cholerae* also encodes two ParE toxins, and these were cloned into a pBAD plasmid with expression driven by arabinose induction. Both VcParE1 and VcParE2 toxins exerted a maximum reduction in *V. cholerae* N16961 cell counts within 4 h of induction (Fig. 3A and C). When compared to the uninduced sample, induction of VcParE2 with 0.2% ara triggered a loss of 5-logs to 6-logs of viable cell growth in the 4–8 h timeframe, while VcParE1 had a 2.8-log to 3-log loss at the same conditions. Control samples harboring the same vector but lacking an inserted gene exhibit a normal growth profile at the arabinose concentrations used (Fig. S1).

Induction of VcParE1 and VcParE2 also significantly increased the mutation frequency (Fig. 3B and E). The VcParE1 toxin induction with 0.02% ara resulted in a 0.5-log increase, while induction at 0.2% further raises this to approx. 1.5-log increase (Fig. 3B). Under the same induction conditions, cultures harboring VcParE2 generate the highest values measured in our study at greater than 5-log increase in mutation frequency (Fig. 3E). While the VcParE1 toxin maintains mutation frequencies below that of the CIP control, the VcParE2 toxin exceeds the action of 0.5× MIC CIP by almost 3-log values. These constructs were also expressed in *E. coli* MG1655 cells, where both VcParE1 and VcParE2 exerted dose-dependent toxicity leading to a maximum reduction of viability within 2 h of induction (Fig. 3C and F). Consistent with the observation in *V. cholerae* N16961 cells, the VcParE2 toxin appeared to exhibit stronger toxicity.

**Fig 4:**
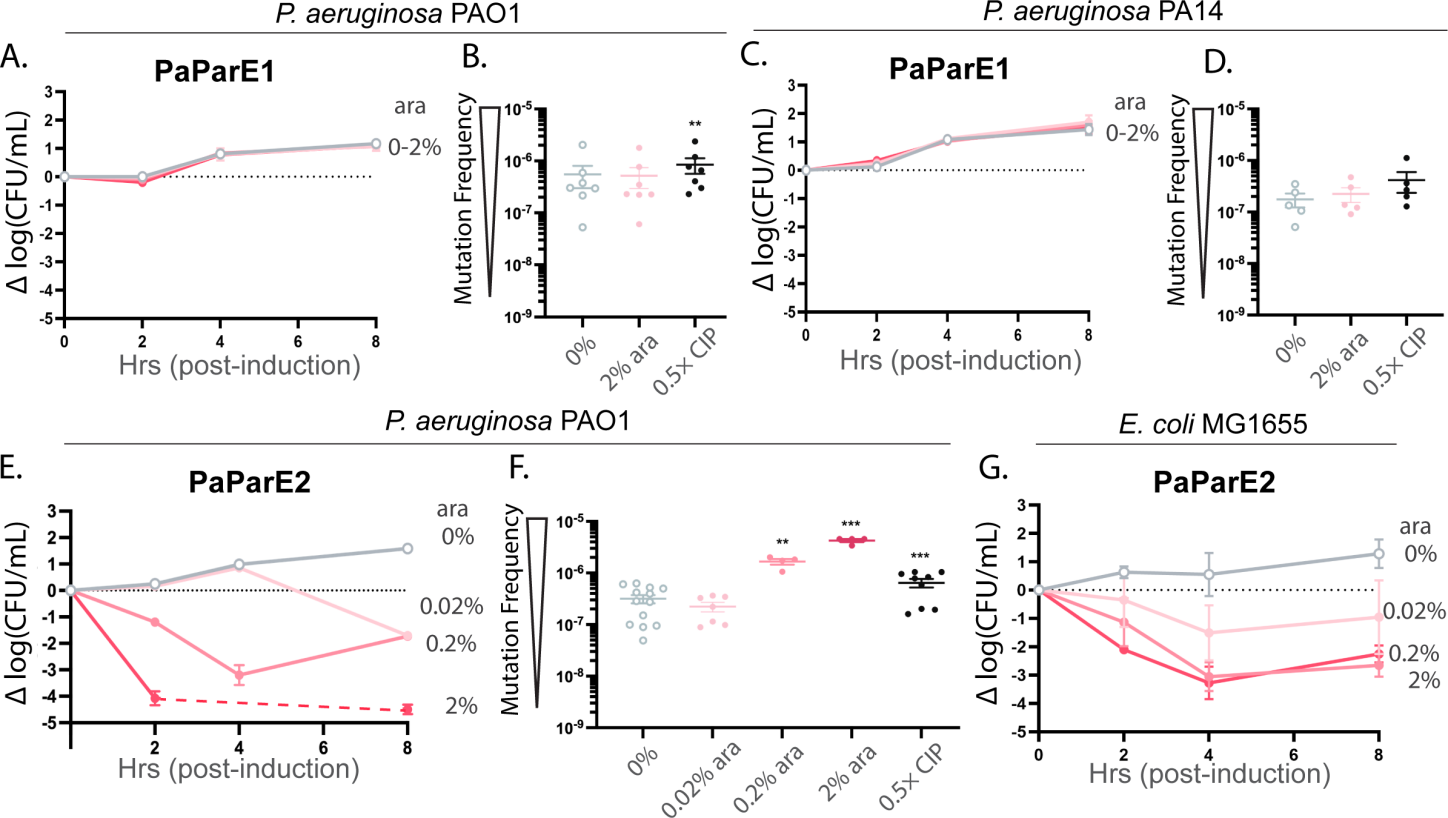
One ParE toxin encoded in the genome of *P. aeruginosa* does not impact the viability of any bacterial strain tested, consistent with a lack of impact on the frequency of mutations, while the ParE2 toxin found in many strains of this bacterium is potently toxic yet has an attenuated impact on mutation frequency. (**A**) Induction of PaParE1 in either the PAO1 or PA14 (**C**) strains has no impact on viability or on mutation frequency (**B, D**). In contrast, the ParE2 toxin is potently toxic to the PAO1 strain (**E**) with values at 4 h below the limit of detection (dashed line), and this induction triggers (**F**) modest increases in mutation frequency. It is of note that the positive control treatment with ciprofloxacin (CIP) also causes only modest impacts to the mutation frequency. (**G**) PaParE2 also displays toxicity in *E. coli* MG1655, although to a lower level than to its native host. Viability data are presented as change from initial CFU/mL (dashed line); all data are displayed as standard error of the mean (SEM) calculated from three independent experiments except for PaParE1 in the PA14 strain, which has two independent replicates that are consistent with the PAO1 results. Mutation frequency of ParE-induced or CIP-treated cells was compared to that of the 0% induction cells using an unpaired two-tailed Student’s *t*-test: **P < 0.01 and ***P < 0.001.

As with *M. tuberculosis* and *V. cholerae*, *P. aeruginosa* encodes two genomic ParDE systems. Of all tested systems, PaParE1 is the only toxin tested that had no impact on viability irrespective of induction strength, including expression in either *P. aeruginosa* PA14 or PAO1 strains (Fig. 4A and C); resulting CFU counts are equivalent to those of the “empty” vector (Fig. S1). In contrast, the PaParE2 toxin exhibited a dose-dependent decline in *P. aeruginosa* PAO1 cell viability (Fig. 4E) with a 0.2% induction level which led to a maximum approx. 3.2-log reduction in cell counts from the starting level within 4 h of induction, while a 2% induction level led to an approx. 5-log reduction in the same time frame. When no arabinose inducer was added, cell counts increased by approx. 1.5-fold (Fig. 4A and E) over the same time period, consistent with the approx. 1.6-fold increase in cell counts observed with the empty vector control (Fig. S1).

It is notable that the PaParE1 toxin correspondingly did not lead to an increase in mutation frequency, even at a relatively high induction of 2% ara (Fig. 4B and D). In contrast, the PaParE2 toxin was able to induce an increase in mutation frequency of 1.2-logs at the same induction strength, with a shallow dose dependence resulting in no change at the lowest tested 0.02% ara (Fig. 4F). The control samples treated with 0.5× MIC of CIP had only a modest increase in mutation frequency of approx. 0.5-log for the PAO1 and PA14 strains, suggesting that gyrase inhibition does not lead to error-prone repair in these strains of *P. aeruginosa*.

The PaParE1 toxin exhibited essentially no toxicity to *E. coli* MG1655 cells (Fig. S2A), while PaParE2 mediated robust dose-dependent toxicity with an approx. 4-log decrease at and beyond 4 h induction (Fig. 4G). Sodium dodecyl-sulfate polyacrylamide gel electrophoresis (SDS-PAGE) of soluble lysate samples followed by Western blotting confirmed robust expression of the PaParE1 toxin protein in cells (Fig. S2B and C, similar results obtained with *P. aeruginosa* cultures, data not shown), indicating that PaParE1’s lack of toxicity is intrinsic rather than due to deficient expression.

### Appending the C-terminal sequence from toxic ParE proteins to non-toxic PaParE1 is not sufficient to impart toxicity

**Fig 5:**
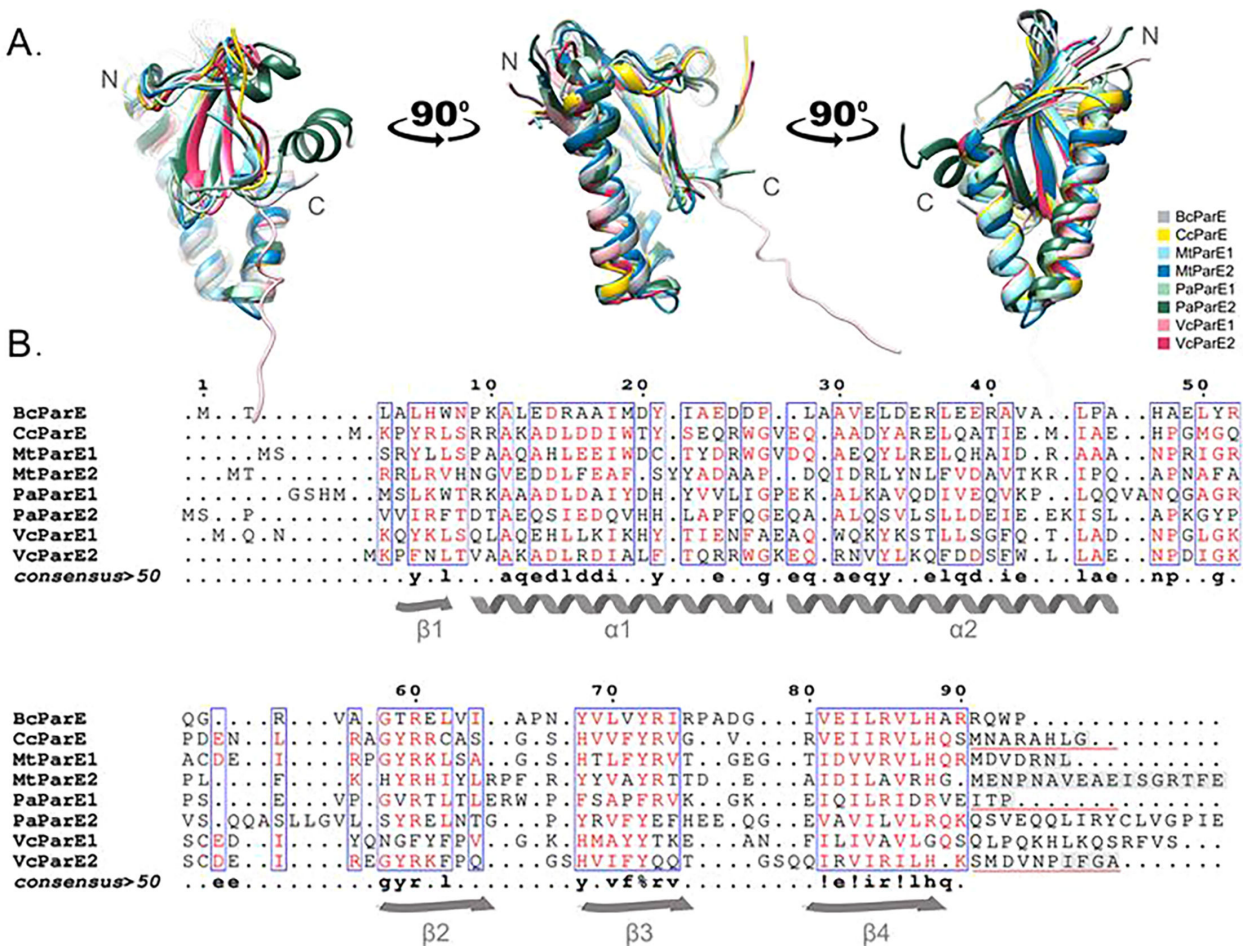
Structural and sequence alignments of ParE toxins tested in the current work. (**A**) The three-dimensional structures of seven ParE toxins were superimposed: BcParE, AlphaFold model AF-B4EF23-F1-model_v4; CcParE, PDB 3KXE (50); MtParE1, PDB 8C24 (15); MtParE2, PDB 8C26 (15); PaParE1, PDB 6XRW (49); PaParE2, AlphaFold model AF-Q9I5J9-F1-model_v4; VcParE1, AlphaFold model AF-O68848-F1-model_v4; VcParE2, PDB 7R5A (35). Note that all crystallographic structures are pictured without the bound ParD antitoxin; further, the C-terminal regions are not complete in those structures (shaded gray boxes, panel B). (**B**) Structure-based aligned sequences with secondary structures indicated (ESPript [55]). Residues with a similarity score exceeding 50% (4 out of 8) are deemed highly similar and are colored in red and framed in blue; amino acids omitted from crystallographic structures due to disorder are indicated by gray shaded boxes. C-terminal sequences used for construction of chimeric ParE toxins are underlined in red. Consensus is listed below, % = F or Y, ! = I or V.

Given the high structural conservation coupled with a relatively low sequence conservation (Fig. 5) (17), it remains unknown how ParE toxins interact with gyrase to mediate its inhibition, although the lack of toxicity of PaParE1 suggests it may have a hallmark difference. We and others have noted that the superposition of ParE toxin structures highlights some variability in the placement of the C-terminus (Fig. 5A), which in all experimentally determined structures interacts with antitoxin and with the most terminal amino acids too disordered to place into electron density (Fig. 5B, gray shaded boxes) (15, 50–52). AlphaFold models for ParE toxins, in the absence of the partner antitoxins, frequently depict the C-termini as forming an alpha-helix (e.g., as shown in Fig. 5A for PaParE2) (53), and we and others have suggested that this secondary structure may play a role in interactions with DNA gyrase (14, 15). Several studies demonstrate that mutations of specific residues or truncations of the C-terminal region decrease ParE toxicity (31, 32, 54). Of specific relevance are reports for ParE toxins from *C. crescentus* or MtParE2 that lost toxicity as the unstructured C-terminus was truncated, although this may also be indirect by altering the stability of the truncated ParE toxin (35). The PaParE1 toxin has the shortest unstructured C-terminus (Fig. 5B) and exerts no toxicity unless cells have an impaired recombination repair system (e*.*g., NovaBlue strain of *E. coli*) (16).

**Fig 6:**
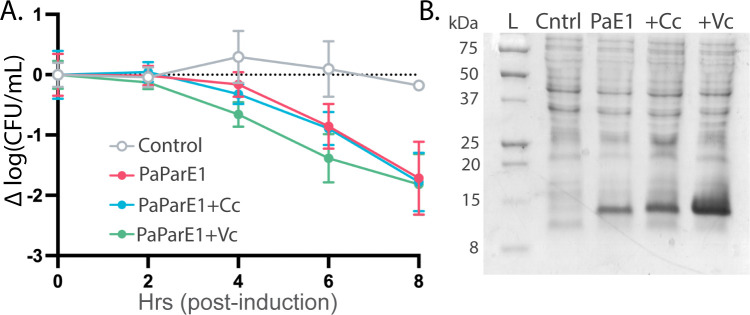
The amino acid sequence of ParE C-termini does not impact toxicity but can enhance expression. (**A**) *E. coli* cells transformed with a pET15b expression vector (control, gray line), or this vector harboring the coding sequence for PaParE1 (magenta line), PaParE1 with the C-terminus from the *C. crescentus* ParE toxin (+Cc, blue line) and with the C-terminus from VcParE2 (+Vc, green line) were induced and viability monitored (data presented as in Fig. 1). No significant differences in viability as a function of C-terminal sequence are observed. (**B**) The expression of each ParE toxin was assessed by electrophoresis, and based on the intensity of staining with Coomassie dye, the levels are comparable to improved with the changed C-terminal sequences.

As PaParE1 is essentially non-toxic, as well as contains the shortest C-terminus, it is an ideal model to test the contributions to toxicity of the ParE C-terminal sequences. The C-terminus of the VcParE2 toxin and a ParE toxin from *C. crescentus* were selected, and the region from immediately after the last beta strand to the C-terminus of the toxin was appended to PaParE1 (Fig. 5B, red underlined amino acids), yielding two chimeric versions. These were transformed to a *recA^−^ E. coli* strain (NovaBlue) to maximize potential toxicity, and expression was induced. Switching the C-terminus of PaParE1 did not impart any appreciable difference to viability (Fig. 6A), with expression of PaParE1 and the two chimeric toxins inducing an approximately 1.7-fold decrease in cell counts as compared to the control sample. Furthermore, the expression level of each ParE toxin increased as the C-terminus was changed, as determined by SDS-PAGE analysis, suggesting that protein stability was not impacted (Fig. 6B). Overall, these results suggest that the amino acid sequences at the C-terminus of ParE toxins do not directly correlate with the observed differences in toxicity levels.

### Antibiotic susceptibility is unchanged despite increased mutation frequency

We questioned if the results from the fluctuation assays would correlate to changes in MIC values for antibiotics and selected some including those commonly used for treating infections caused by the native host bacteria. We also included anti-gyrase antibiotics to assess potential impacts from the shared mechanism of action. Cultures were treated as those used in the fluctuation assay, and where feasible, the same cultures were used to establish MIC values using impregnated strips or disks (Table 2; Fig. S3 to S6). There were significant challenges in re-growing cultures exposed to toxic ParE proteins, and for some, this required normalization of the input cell CFU to that of the uninduced control sample to avoid an inoculation effect. Overall, these assays reveal no increases in MIC values, indicating that the measured mutation frequencies do not confer any increased antibiotic resistance. Of interest are situations of collateral sensitivity, such as for *E. coli* treated with meropenem or trimethoprim, and *B. cenocepacia* treated with the piperacillin-tazobactam combination. It is interesting to note that anti-gyrase quinolone antibiotics are still effective, suggesting that ParE toxins are not providing target protection in these cells.

**TABLE 2:** MIC values as a function of ParE induction^*a*^*^,c^*

BcParE/*B. cenocepacia*	MPN	PIP/Tazo	LEV
0% rhm	4–8	**>256**	6
0.0005%	8	**65–128**	6
0.002%	6–8	**16–96**	3–6

MtParE1/*E. coli*	MPN (10 µg)^*b*^	TMP (5 µg)^*b*^	CIP
0% ara	**33.4–34.6**	**25.4–27.2**	0.008
0.02%	**38.7–39.8**	**31.0–35.1**	0.004
0.2%	**41.1–41.8**	**33.0–35.6**	0.004
2%	**41.3–41.6**	**35.3–35.8**	0.004–0.006

MtParE2/*E. coli*	MPN (10 µg)^*b*^	TMP (5 µg)^*b*^	CIP
0% ara	**28.5–29.5**	**25.0–25.6**	0.006
0.00001%	**27.5–33**	**24.6–28.5**	0.006
0.001%	**33.5–36.0**	**30.9–33.0**	0.004–0.006

PaParE1/*P. aeruginosa*	MPN (10 µg)^*b*^	TOB (10 µg)^*b*^	LEV
0% ara	30.7–30.9	20.6–23.3	0.19–0.25
2%	30.7–30.9	21.4–23.2	0.19–0.25

PaParE1/*P. aeruginosa*	MPN (10 µg)^*b*^	TOB (10 µg)^*b*^	LEV
0% ara	27.8–28.0	21.8–21.9	0.25–0.38
2%	27.6–28.0	22.0–22.3	0.25–0.38

PaParE2/*P. aeruginosa*	MPN (10 µg)^*b*^	TOB (10 µg)^*b*^	LEV
0% ara	4–8	26.0–26.3	0.125
0.2%	6–8	25.5–25.6	0.125
2%	6–8	25.8–25.9	0.125

VcParE1/*V. cholerae*	AZM	DOX	LEV
0% ara	0.38–2	0.19–0.25	0.008
0.02%	0.5–2	0.19–0.25	0.012–0.016
0.2%	0.38–2	0.19–0.38	0.008–0.024

VcParE2/*V. cholerae*	AZM	DOX	LEV
0% ara	0.38–0.75	0.38	0.006–0.032
0.02%	0.38–0.75	0.094	0.008–0.012
0.2%	0.75–1	0.064–0.25	0.004

^*a*^ Each value is based on at least two independent measurements; entries in bold exhibit a clear change in susceptibility, defined as more than two twofold MIC changed or the range of disk area changed, as a function of ParE induction.

^*b*^ Antibiotic tested in disk form; values for diameter of clearing listed. Data not listed for RIF because there were no MIC change for cultures tested*.* Similarly, *P. aeruginosa* strains were tested with AZM and PIP; no MIC changes were noted.

^*c*^ AZM, Azithromycin; PIP, Piperacillin; DOX, Doxycycline; LEV, Levofloxacin.

## DISCUSSION

Chromosomal TA systems are intriguing with respect to potential functions (11), including the possibility for “self-intoxication” to elicit loss of growth or viability, or potentially to protect the target against other insults. This could lead to altered growth states, such as viable but non-culturable or persister cells (55–57); however, the extent of toxicity, and thus potential for cell recovery, will be toxin-specific. We sought to extend our previous study of chromosomal ParE toxins to understand how exposure in their native host strains could contribute to altered growth and potentially contribute to survival by promoting genetic changes leading to antibiotic resistance (16). Our studies reveal a stagnation in growth or loss of viability for all tested except the previously noted non-toxic ParE1 from *P. aeruginosa* (Fig. 4A and C), despite its robust expression level (Fig. S2) (16). For all other ParE toxins, impacts on growth were directly correlated to induction strength, although rarely was viability completely abrogated. Most ParE toxins tested were able to reduce viability by at least 99% (2-logs) by 4 h post-induction, with many reducing growth by 3-log to 4-log values. Attempts to capture expression levels for toxins other than PaParE1, such as through Western blotting, mass spectrometry, and biolayer interferometry, were unsuccessful due to a lack of sensitivity of the methods for the low levels of expressed proteins (data not shown). This leads us to conclude that ParE toxins are impressively effective in altering bacterial growth.

While the source of the varied toxicity profiles for ParE toxins remains an open question, and we cannot exclude different absolute expression levels, increasingly it appears that the source may lie within ParE sequence differences. Several studies suggest that mutation of specific residues within, or truncations of, the C-terminus decreases ParE toxicity (15, 54, 58, 59). To date, all experimentally determined structures of ParE are in complex with ParD antitoxins, where this C-terminal region adopts a coil-like secondary structure or is too mobile to be visualized (Fig. 5) (15, 50–52). The AlphaFold algorithm predicts that this region adopts a helical conformation (53), although this may be a bias from relying on the closely related RelE toxins that contain a C-terminal helix. Furthermore, while PaParE1 is robustly expressed (Fig. S2), it is not toxic and has the shortest C-terminus among ParE toxins (Fig. 5). The BcParE toxin has the next shortest C-terminus with only one additional amino acid relative to PaParE1. Two of the most toxic ParE proteins, MtParE2 and PaParE2, contain the longest C-termini with 17 amino acids. Overall, these observations support the suggestion that the C-terminus of ParE toxins is required for toxicity and, given the variations in their sequences, may contribute to differences in the extent of toxicity. We have directly tested this hypothesis by constructing chimeric ParE toxins with C-terminal amino acids appended to the non-toxic PaParE1 scaffold. While these chimeras express as well or better than the PaParE1 toxin, there is no gain of toxicity. Therefore, it appears that while the C-terminus may impact potency, it is not sufficient to impart toxicity to the PaParE1 toxin. Furthermore, we cannot rule out a role for the C-terminal regions in improving expression or solubility, and this explanation is supported by our data. An interesting future direction may be the expression of just the C-terminus, if it remains soluble, to gauge the potential for an independent role in gyrase inhibition.

The ParE toxin family phenocopies other gyrase poisons, including antibiotic treatments, leading to the accumulation of double-stranded DNA breaks that activate the SOS pathway (8, 60, 61). This bacterial stress response mediates mutagenic repair and is implicated in increased resistance, horizontal gene transfer, and activation of resident prophages (36, 56, 62, 63). Mutation frequencies can be measured in laboratory settings by following the classic Luria-Delbrück fluctuation assay (28). Our approach refined this standardized procedure to evaluate the frequency of mutations arising after induction of ParE toxins by defining an exposure timepoint relative to the viability measurements. Additional adjustments were required to ensure recovery of sufficient numbers of surviving cells to allow accurate measurement of rare mutants, with frequencies typically starting on the order of 10 cells per billion (a frequency of 1 × 10^−8^) and increasing up to as many as 8 cells per one thousand (a frequency of 8 × 10^−3^). We find a striking induction-dependent increase in mutation frequency that correlates with increased toxicity, including a lack of mutagenesis for the non-toxic PaParE1, and the largest increases for VcParE2 and MtParE2 (Fig. 2 and 3). The tested *P. aeruginosa* strains treated with anti-gyrase antibiotic CIP at 0.5× the MIC have only modestly increased mutation frequencies. The BcParE toxin is among the most increased with respect to the mutation frequency by a factor of approx. 10,000.

The last series of experiments was devised to assess if these increases in mutation frequency would also result in increased MIC values for antibiotics. While mutations were detected by selection of colonies resistant to RIF and TMP, which are antibiotics, this resistance is well characterized to arise from single specific point mutations (28). We find that the mutagenesis initiated by the presumably random location of DNA breaks resulting from ParE-mediated inhibition was unable to produce the more complex changes needed to yield resistance to commonly used antibiotics. Collateral sensitivity was noted for selected antibiotics tested against *E. coli* and *B. cenocepacia*, such that the MIC values for MRP, TMP, and PIP/Tazo decreased as ParE induction increased. These data support the conclusion that bacterial cells die from ParE-mediated breaks before repair can allow specific mutations to accumulate into functional antibiotic resistance. Overall, if the ParE-mediated molecular mechanism can be harnessed, our data support that it would be a useful antibacterial approach with no anticipated adverse impacts on current antibiotic use.

## MATERIALS AND METHODS

### Structure comparison and sequence alignments

Structure-based alignments were carried out using UCSF Chimera (64) and formatted using ESPript 3.0 (65). For BcParE and PaParE2, AlphaFold models were used as no experimentally determined structures are available (53).

### Molecular biology and bacterial stock generation

Bacterial strains, plasmids, and gene identifiers are listed in Table 3 and Table S1.

**TABLE 3:** Bacterial strains and plasmids used in this study

	Description	Source of reference
Strains		
*Escherichia coli* K-12		
MG1655	F^-^ lambda^-^ *ilvG^-^ rfb*-50 *rph*-1, experimental strain	In-house lab collection
NovaBlue	Experimental strain with *recA*, *endA* mutations	Novagen
*Pseudomonas aeruginosa* (Pa)		
PA14	Experimental strain	Dr. Erike Lutter (Oklahoma State University)
PAO1	Genomic DNA source, experimental strain	Dr. Erike Lutter (Oklahoma State University)
*Mycobacterium tuberculosis* (Mt)		
Rv1960c	Genomic DNA source	DNA directly purchased from ATCC
*Vibrio cholerae* (Vc)		
N16961	Genomic DNA source, experimental strain	Obtained through BEI Resources, NIAID, NIH (NR-147; ATCC 39315)
*Burkholderia cenocepacia* (Bc)		
J2315	Genomic DNA source, experimental strain	Obtained through BEI Resources, NIAID, NIH (NR-701; ATCC BAA-245)
*Caulobacter crescentus* (Cc)	Genomic DNA source	Dr. Sean Crosson (Michigan State University)
Plasmids		
pHerd20T	ColE1/pMB1/pBR322/pUC origin of replication, pBAD promoter, acceptor of Pa ParDE genes, Amp^R^, donor of *araC*-P_BAD_ fragment	Dr. Hongwei Yu (Marshall University)
pMind	ColE1/pMB1/pBR322/pUC origin of replication, tetRO promoter, Neo^R^ Kam^R^	Brian Robertson (Addgene plasmid # 24730; http://n2t.net/addgene:24730; RRID:Addgene_24730)
pMindBAD	pMind tetRO promoter replaced with a 1.216 kb fragment of *araC*-P_BAD_ from pHerd20T, acceptor of Mt ParDE genes	This study
pBAD33	p15A origin of replication, araBAD promoter, Cm^R^, acceptor of Vc ParDE genes	Dr. Matthew K. Waldor (Harvard University)
pSCrhaB2	pBBR1 origin of replication, rhamnose promoter, acceptor of Bc ParDE genes, Tmp^R^	Miguel Valvano (Addgene plasmid # 113634; http://n2t.net/addgene:113634; RRID:Addgene_113634)
pET-15b	T7 promoter, Amp^R^, acceptor of PaParE1 genes, retains N-terminal 6× His affinity tag with thrombin cleavage site	Invitrogen

DNA sequences of *parDE* operons were obtained from the TADB (4). Primers were designed with the NEBuilder tool (https://nebuilder.neb.com/#!/) (listed in Table S3) to amplify genes from chromosomal DNA for incorporation into appropriate plasmids using HiFi DNA assembly (NEB). Chimeric PaParE1 constructs were generated by amplifying the C-terminal coding regions of *C. crescentus parE1* or *V. cholerae parE2* and appending to Pa *parE1* in a pET-15b plasmid using Q5 mutagenesis reactions (NEB). The N-terminal appended 6× His affinity tag found in this construct of PaParE1 has previously been validated as having no impact on potential toxicity or inhibition of DNA gyrase (16, 50). Resulting plasmid constructs were extracted and purified using Zyppy Plasmid Miniprep Kits (Zymo Research) and verified by DNA sequencing from Genewiz or Plasmidsaurus. Constructs generously donated by other groups (Table 3) were also verified by sequencing before use.

Plasmids were transformed into competent cells by heat shock (for *E. coli* strain) or electroporation (for other strains) methods according to standard protocols. Successful transformation of *V. cholerae* required allowing electroporated cells to incubate for 3 h at 37°C without shaking prior to further selection or growth. Transformed cultures were propagated from individual colonies and stored as LB-20% glycerol stocks at −80°C.

### Viability assays

Cultures were inoculated from experimental strains (−80°C frozen 20% glycerol stocks) into Luria-Bertani (LB) media, supplemented with appropriate antibiotics and 1% glucose, and grown overnight (18–20 h) at 37°C with shaking at 200 rpm. The overnight cultures were subsequently diluted at a 1:20 ratio in fresh media (LB for *E. coli* strain, M9 minimal media for other bacteria), supplemented with appropriate antibiotics and inducers, and grown at 37°C with shaking at 200 rpm. Induction strengths for *V. cholerae* were limited to a maximum of 0.2% to avoid a previously noted toxicity at higher arabinose concentrations (66). Aliquots of cultures were collected at the defined time intervals, and 10-fold serial dilutions were prepared in sterile 0.9% saline solution or test medium. These were spotted onto LB or M9 agar plates supplemented with appropriate antibiotics and 1% glucose for determination of the CFUs. CFUs were subsequently converted to CFU/mL based on the initial spotting volumes. The limit of detection for CFU/mL measurements was calculated using the following formula: 1 CFU divided by the initial spotting volume (μL), multiplied by 1,000 µL/mL. These assays were performed in at least triplicate (with technical replicates) using unique overnight cultures inoculated from the same glycerol stock. Antibiotics were added for plasmid selection, as appropriate: carbenicillin, 100 µg/mL; kanamycin, 50 µg/mL; chloramphenicol, 25 µg/mL; trimethoprim, 600 µg/mL.

Figures were generated using GraphPad Prism 8.01.

### Minimum inhibitory concentration assays

MICs of CIP, TMP, and RIF were determined using the broth microdilution method with serial dilutions of antibiotics in 96-well microtiter plates (67). Overnight cultures were diluted to 10^6^ CFU/mL (2× bacterial inoculum) in test media and added to the appropriate wells. These were sealed and incubated at 37°C overnight, after which a visual reading was performed to determine the MIC, defined as the lowest antibiotic concentration in which there was no visible bacterial growth. These assays were performed in at least duplicate using unique overnight cultures inoculated from the same glycerol stock.

### Mutation frequency

Overnight cultures were inoculated and grown as described in the viability assay. These were diluted 1:10,000 in test media and grown at 37°C with shaking at 200 rpm to an optical density of 0.2. Cultures were subsequently divided into aliquots, and inducers at specified concentrations were chosen based on the viability assays with the reasoning that the first timepoint after maximum loss of viability may represent the most likely point for the emergence of mutants, or 0.5× MIC CIP was added to individual aliquots. These were subsequently incubated at 37°C 200 rpm for 8 h, except *B. cenocepacia*, which was grown for 15 h due to its slower doubling time (approx. 3 h). Cultures were pelleted and resuspended in defined volumes of sterile saline solution or test medium to ensure sufficient viable mutant cells would be present. Appropriate volumes of the cell resuspensions were then plated on solid LB or M9 agar plates supplemented with plasmid selection antibiotics as well as RIF or TMP at 3–5× MIC to select for mutants. Total viable cell counts were also determined (CFU values) by plating cell resuspensions on solid LB or M9 agar plates supplemented with plasmid selection antibiotics with no RIF or TMP. The mutation frequency was calculated by the ratio of mutant counts per plated volume unit to total viable cell counts per plated volume unit. These assays were performed in at least triplicate (with technical replicates) using unique overnight cultures inoculated from the same glycerol stock.

Figures were generated using GraphPad Prism 8.01.

### Antibiotic susceptibility assays

Cultures were prepared as described for the mutation frequency assay, and in most cases, the same culture was used. Appropriate volumes of cultures were then swabbed onto LB or M9 agar plates, as appropriate, supplemented with plasmid selection antibiotics and lacking inducers. Disks or E-test strips of specified antibiotics were placed onto the agar plates and incubated at 37°C overnight (18–24 h). E-test strips: (BioMerieux) levofloxacin, 32 µg/mL; doxycycline, 256 µg/mL; azithromycin, 256 µg/mL; piperacillin/tazobactam, 256 µg/mL; piperacillin, 256 µg/mL (Liofilchem) meropenem, 256 µg/mL; rifampicin, 256 µg/mL. Disks were generated in-house by spotting a set volume of a known concentration onto a sterile disk (Hardy Scientific), allowing the disk to dry, and then carefully placing it onto the swabbed agar plate. Antibiotic susceptibility was evaluated by assessing the size of the clear zone around the disk or by determining the MIC value based on the scale imprinted on the strips and changes in these values as a function of ParE induction. These assays were performed in at least duplicate with technical replicates using unique overnight cultures inoculated from the same glycerol stock. We note that “clear changes” are defined as MIC values differing by more than two twofold changes and disk diffusion values with less than 20% overlap in diameter measurements.

### Gel electrophoresis and Western blotting techniques

Cell cultures were collected at designated timepoints, and cells were lysed by BugBuster Protein Extraction Reagent (Sigma-Aldrich) according to the manufacturer’s instructions. The resulting cell lysates were collected by centrifugation and mixed with SDS loading dye. Samples were heated at 95°C for 5 min and loaded onto 12% acrylamide gels with a Tris-Tricine buffer system. Electrophoresis was performed to separate protein species, and the protein bands were visualized via Coomassie blue staining. To visualize the protein bands using Western blot, after electrophoresis, samples were transferred to nitrocellulose blotting membrane (Cytiva) using the semi-dry transfer method conducted in the trans-blot turbo transfer system (Bio-Rad). The membrane was blocked with 1% skim milk for 1 h at room temperature and then washed with 1× Tris-buffered saline (TBS) solution containing 0.1% Tween-20 three times. The membrane was then incubated with Alexa Fluor anti-6× His tag antibody (Thermo Fisher) at room temperature for 1 h and washed with 1× TBS solution containing 0.1% Tween-20 three times. The protein bands were visualized using a Chemi-Doc unit.

## SUPPLEMENTAL MATERIAL

Tables S1 to S3; Figures S1 to S6 jb.00416-24-s0001.pdf Table S1: Information for ParE toxins used in this study. Table S2: Details and growth parameters for bacterial studies. Table S3: Primer sequences. Figure S1: Empty Vector expression controls have no impact on bacterial cell viability. Figure S2: PaParE1 toxin is robustly expressed but lacks toxicity. Figure S3: Impact of BcParE toxin expression on *B. cenocepacia* cell antibiotic susceptibility. Figure S4: Impact of PaParE1 and PaParE2 toxin expression on *P. aeruginosa* PAO1 or PA14 cell antibiotic susceptibility. Figure S5: Impact of MtParE1 and MtParE2 toxin expression on *E. coli* MG1655 cell antibiotic susceptibility. Figure S6: Impact of VcParE1 and VcParE2 toxin expression on *V. cholerae* N16961 cell antibiotic susceptibility.

ASM does not own the copyrights to Supplemental Material that may be linked to, or accessed through, an article. The authors have granted ASM a non-exclusive, world-wide license to publish the Supplemental Material files. Please contact the corresponding author directly for reuse.
