# Supplementary material for: Broken beyond repair: TA system ParE toxins mediate effective gyrase inhibition without driving resistance
Source: J Bacteriol. 2025 Mar 3;207(3):e00416-24. doi: 10.1128/jb.00416-24 (PMC11925232; doi:10.1128/jb.00416-24)
Supplement: Tables S1 to S3; Figures S1 to S6 — Table S1: Information for ParE toxins used in this study. Table S2: Details and growth parameters for bacterial studies. Table S3: Primer sequences. Figure S1: Empty Vector expression controls have no impact on bacterial cell viability. Figure S2: PaParE1 toxin is robustly expressed but lacks toxicity. Figure S3: Impact of BcParE toxin expression on B. cenocepacia cell antibiotic susceptibility. Figure S4: Impact of PaParE1 and PaParE2 toxin expression on P. aeruginosa PAO1 or PA14 cell antibiotic susceptibility. Figure S5: Impact of MtParE1 and MtParE2 toxin expression on E. coli MG1655 cell antibiotic susceptibility. Figure S6: Impact of VcParE1 and VcParE2 toxin expression on V. cholerae N16961 cell antibiotic susceptibility. [file jb.00416-24-s0001.pdf]

Supplemental Information

Title:

Broken beyond repair: TA system ParE toxins mediate effective gyrase inhibition without driving resistance

Authors:

Chih-Han Tu[1†], Shengfeng Ruan[1, 2†], Michelle Holt[1,3], Christina R. Bourne[1*]

†: Equivalent contributions to authorship

*: Corresponding author (cbourne@ou.edu)

1: Department of Chemistry and Biochemistry, University of Oklahoma, Norman, OK 73019, USA
2: Current address (SR): BioNTech, Cambridge, MA
3: Current address (MH): KCAS Bio LLC, Olathe, KS

Table S1. Information for ParE toxins used in this study.

Table S2. Details and growth parameters for bacterial studies.

Table S3. Primer sequences.

Figure S1. Empty Vector expression controls have no impact on bacterial cell viability.

Figure S2. PaParE1 toxin is robustly expressed but lacks toxicity.

Figure S3. Impact of BcParE toxin expression on *B. cenocepacia* cell antibiotic susceptibility.

Figure S4. Impact of PaParE1 and PaParE2 toxin expression on *P. aeruginosa* PAO1 or PA14 cell antibiotic susceptibility.

Figure S5. Impact of MtParE1 and MtParE2 toxin expression on *E. coli* MG1655 cell antibiotic susceptibility.

Figure S6. Impact of VcParE1 and VcParE2 toxin expression on *V. cholerae* N16961 cell antibiotic susceptibility.

Table S1. Information for ParE toxins used in this study

Native bacterial host(s)	Abbreviation	Vector	Gene Annotation	UniProt ID
B. cenocepacia J2315*	BcParE	pSCrha2	QU43_RS36845	B4EF23
M. tuberculosis H37Rv	MtParE1	pMindBAD	Rv1959c	P9WHG7
M. tuberculosis H37Rv	MtParE2	pMindBAD	Rv2142c	P9WHG5
P. aeruginosa PAO1, PA14	PaParE1	pHerd20T	PA0124, PA14_01510	Q9I708, Q02UV4
P. aeruginosa PAO1	PaParE2	pHerd20T	PA0729	Q9I5J9
V. cholerae N16961**	VcParE1	pBAD33	VC_RS14830	O68848
*V. cholerae*N16961**	VcParE2	pBAD33	VC_RS15105	Q9KMJ0

Table S2. Details and growth parameters for bacterial studies

	**Approx. doubling time**	**Media for assays**	**Fluctuation Assay**	
			Dilution	***Antibiotic***
B. cenocepacia J2315	3 hr	M9	1:10K	RIF
E. coli MG1655	18 min	LB	1:10 K	RIF
P. aeruginosa PA14	78 min	M9	1:10 K	TMP
P. aeruginosa PAO1	78 min	M9	1:10 K	TMP
V. cholerae N16961	23 min	M9	1:10 K	RIF

Table S3. Primer sequences

Target Amplified	Primer Sequence
Bc *parE* (gene),<br>pSCrhaB2 (vector)	*Gene FWD:* ATCCTCTAGAATGACCCTGGCGCTGCAC<br>*Gene REV:* AGCCAAGCTTTTACGGCCATTGCCGTCG<br>*Vector FWD:* ATGGCCGTAAAAGCTTGGCTGTTTTGGC<br>*Vector REV:* CCAGGGTCATTCTAGAGGATCCCCGGGTAC
ara operon<br>(gene),<br>pMIND (vector)	*Gene FWD:* TAGAAATAATCGTGGAAGGAGGAGAGGATCCATG<br>*Gene REV:* GTTGTCATAATCTAGAGGTACCGAGCTC<br>*Vector FWD:* TACCTCTAGATTATGACAACTTGACGGC<br>*Vector REV:* TCCTTCCACGATTATTTCTAGCCCAAAAAAAC
Mt *parE1* (gene),<br>pMindBad (vector)	*Gene FWD:* GTGGAATCCTGACAGGATCCGTGAGTAGCCGATACCTTC<br>*Gene REV:* CATACTTAATTAAGGATATCTCAGAGGTTCCGGTCGAC<br>*Vector FWD*: GATATCCTTAATTAAGTATGCATCG<br>*Vector REV:* GGATCCTGTCAGGATTCC
Mt *parE2* (gene)<br>(with N-Strep),<br>pMindBad (vector)	*Gene FWD:* GCAGTTTGAAAAAATGACGCGCAGGCTGCGC<br>*Gene REV:* GGATGGCTCCACATGGATCCTGTCAGGATTCCACGATGAG<br>*Vector FWD*: GATATCCTTAATTAAGTATGCATCG<br>*Vector REV:* GGATCCTGTCAGGATTCC
Pa *parE1* (gene)<br>(with N-6·His),<br>pHerd20T (vector)	*Previously published:* Muthuramalingam M, *et. al*<br>Mol Microbiol. 2019 Feb;111(2):441.
Pa *parE2* (gene)<br>(with N-6·His),<br>pHerd20T (vector)	*Gene FWD:* GCTCGGTACCATGTCCCCGGTCGTCATTC<br>*Gene REV:* AGGATCCCCGTCACTCGATTGGCCCCAC<br>*Vector FWD:* AATCGAGTGACGGGGATCCTCTAGAGTC<br>*Vector REV:* CCGGGGACATGGTACCGAGCTCGAATTC
Vc *parE1* (gene),<br>pBAD33 (vector)	*Previously published: Yuan J, et. al J. Bacteriol. 2010*<br>193(3):611.
Vc *parE2* (gene),<br>pBAD33 (vector)	*Gene FWD:* CGGCGCATAATCTAGAGTCGACCTGCAG<br>*Gene REV:* TTTTAGCCATAACTAGGCTCCTTACTGG<br>*Vector FWD:* CGGCGCATAATCTAGAGTCGACCTGCAG<br>*Vector REV:* ATGGTTTCATAACTAGGCTCCTTACTGG

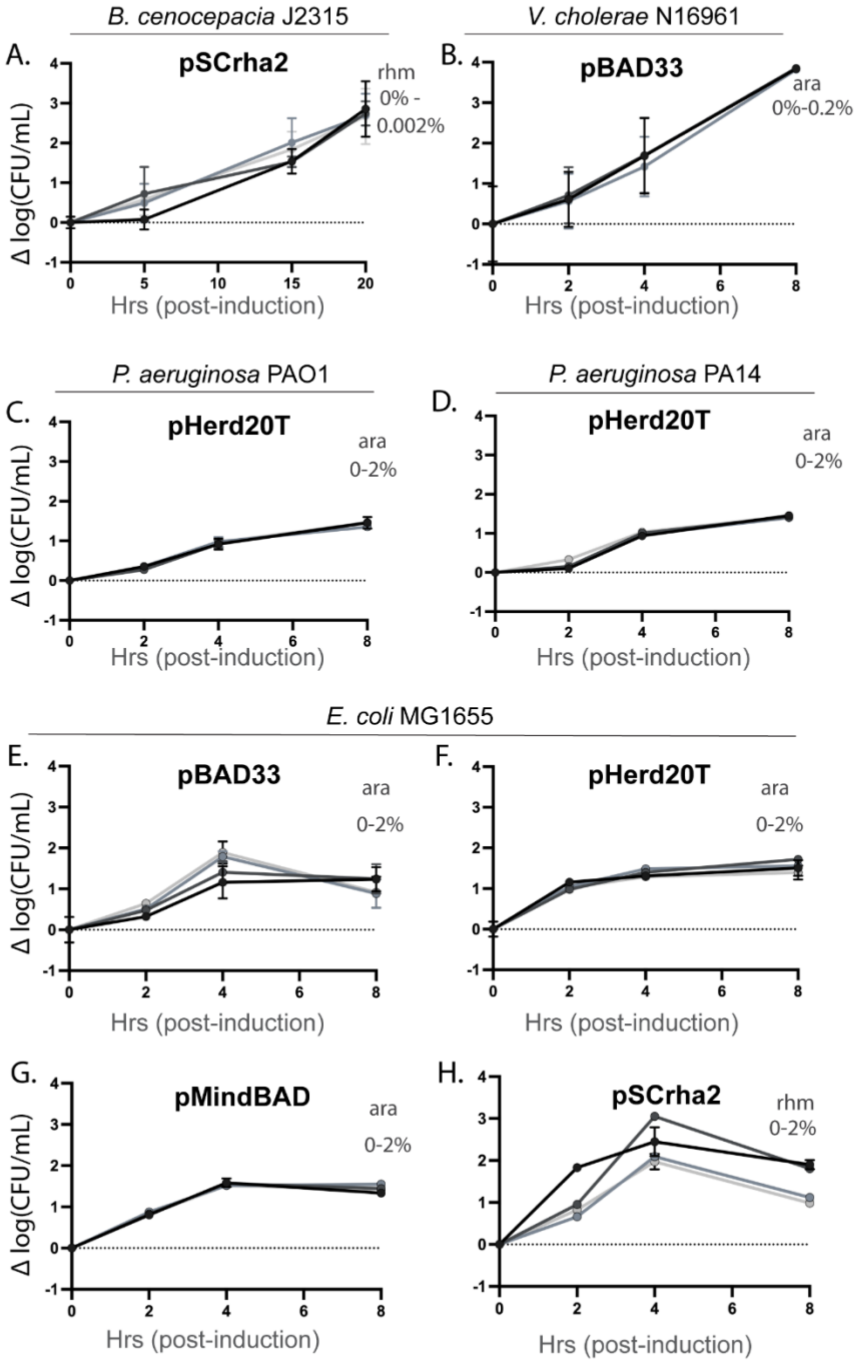

Figure S1. Empty Vector expression controls have no impact on bacterial cell viability. The expression of “empty vectors” (plasmid vectors lacking a toxin, antitoxin, or TA operon in the open reading frame) were tested to ensure they do not induce toxicity upon induction. Samples were tested in parallel with toxin-containing plasmids. Each data point is presented with the standard error of the mean (SEM), representing at least two independent experiments.

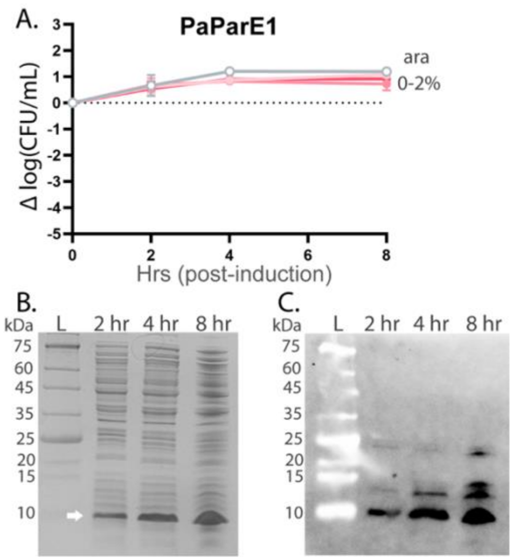

Figure S2. PaParE1 toxin is robustly expressed but lacks toxicity. (**A**) Similar to expression in its native host, PaParE1 has no impact on the viability of *E. coli* MG1655 cells. (**B**) Aliquots of *E. coli* MG1655 cells were removed at the indicated timepoints after induction, electrophoresed, and visualized with Coomassie stain, confirming expression based on the band at approx. 10 kDa (white arrow). (**C**) Western blot to detect the appended 6x His sequence, further confirming the expression of apparently non-toxic PaParE1. *L: protein ladder.*

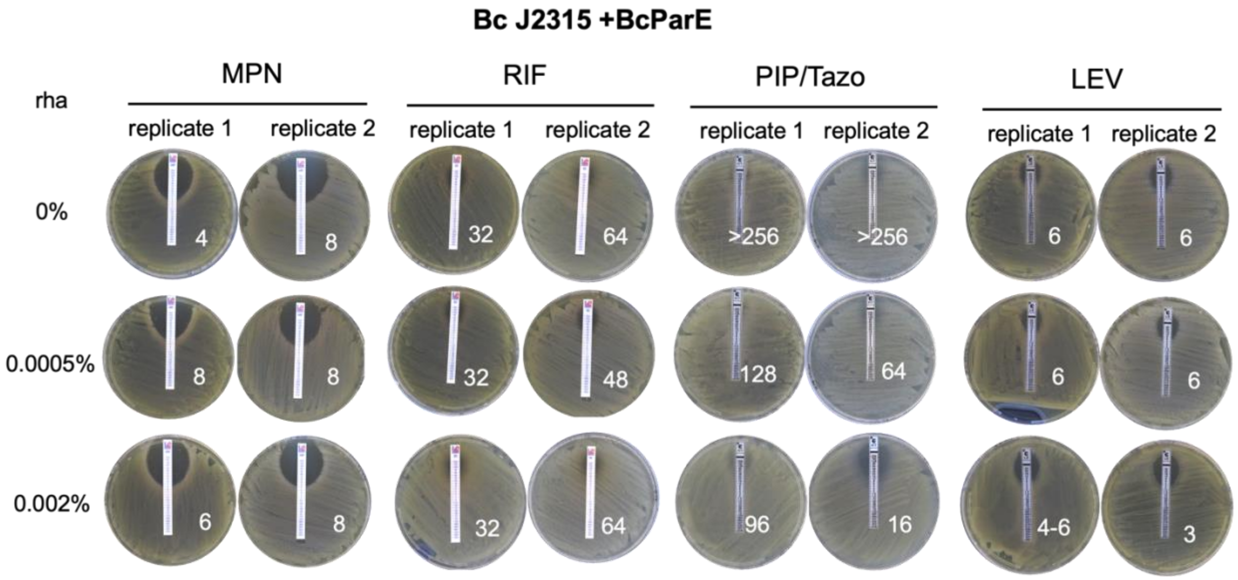

Figure S3. Impact of BcParE toxin expression on *B. cenocepacia* cell antibiotic susceptibility. Cell cultures were swabbed on defined minimal media (M9) plates after 15 hrs of BcParE expression at indicated rhamnose (rha) induction strengths, and then E-strips of specific antibiotics were placed on the plates. Antibiotic susceptibilities were evaluated after 48 hrs growth by determining the limit of cleared growth indicating the MIC value as printed on strips. MRP: meropenem; RIF: rifampicin; PIP/Tazo: piperacillin+tazobactam; LEV: levofloxacin.

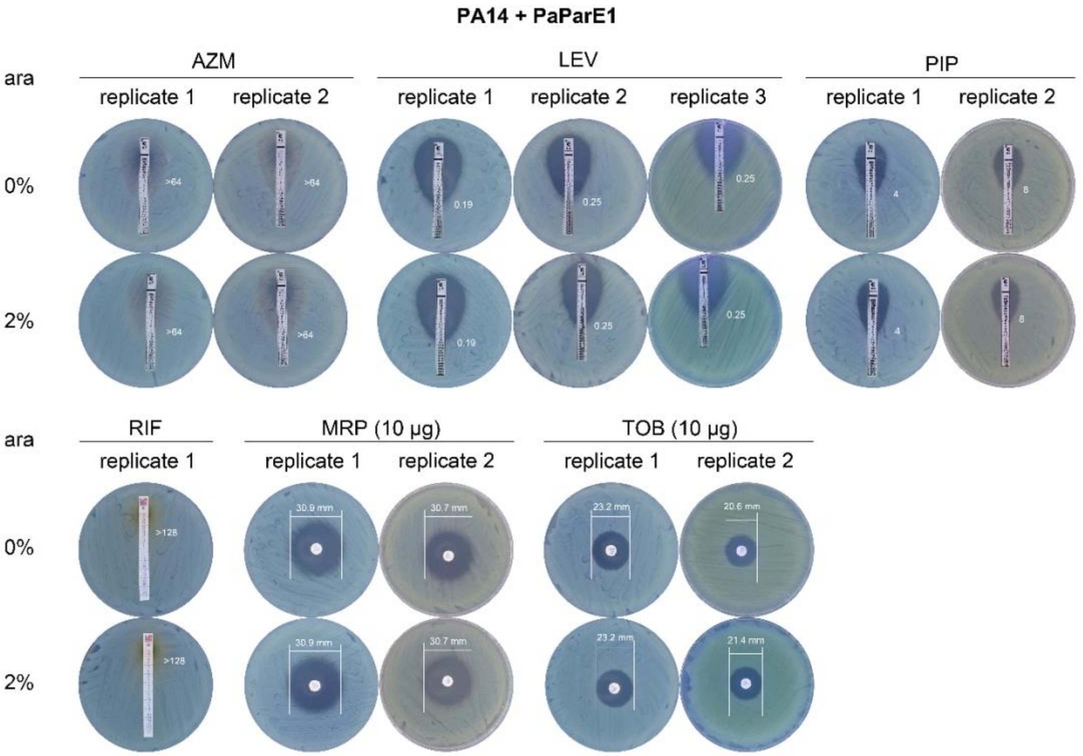
PA14 + PaParE1
ara
AZM
LEV
PIP
replicate 1
replicate 2
replicate 1
replicate 2
replicate 3
replicate 1
replicate 2
0%
2%
>64
>64
0.19
0.25
0.25
4
8
>64
>64
0.19
0.25
0.25
4
8
RIF
MRP (10 µg)
TOB (10 µg)
replicate 1
replicate 1
replicate 2
replicate 1
replicate 2
>128
30.9 mm
30.7 mm
23.2 mm
20.6 mm
>128
30.9 mm
30.7 mm
23.2 mm
21.4 mm

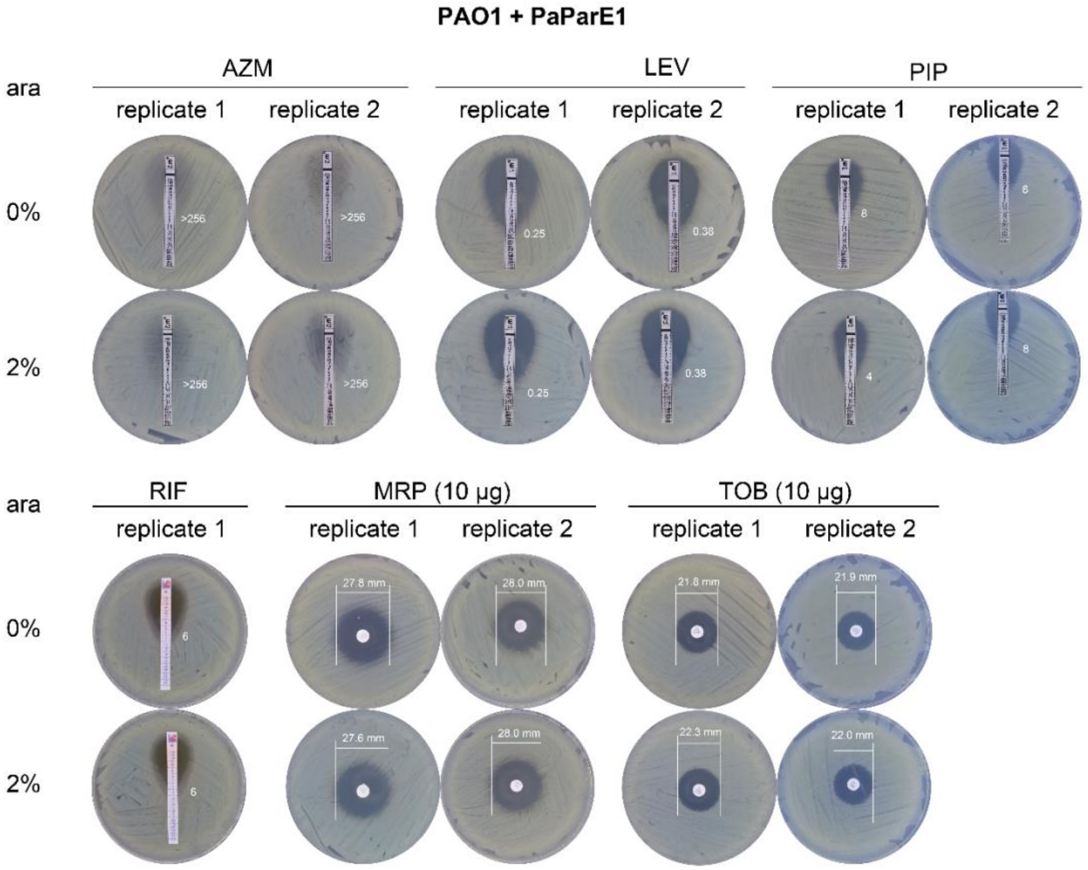
PAO1 + PaParE1
ara
AZM
LEV
PIP
replicate 1
replicate 2
replicate 1
replicate 2
replicate 1
replicate 2
0%
2%
>256
>256
0.25
0.38
8
8
>256
>256
0.25
0.38
4
8
RIF
MRP (10 µg)
TOB (10 µg)
replicate 1
replicate 1
replicate 2
replicate 1
replicate 2
6
27.8 mm
28.0 mm
21.8 mm
21.9 mm
6
27.6 mm
28.0 mm
22.3 mm
22.0 mm

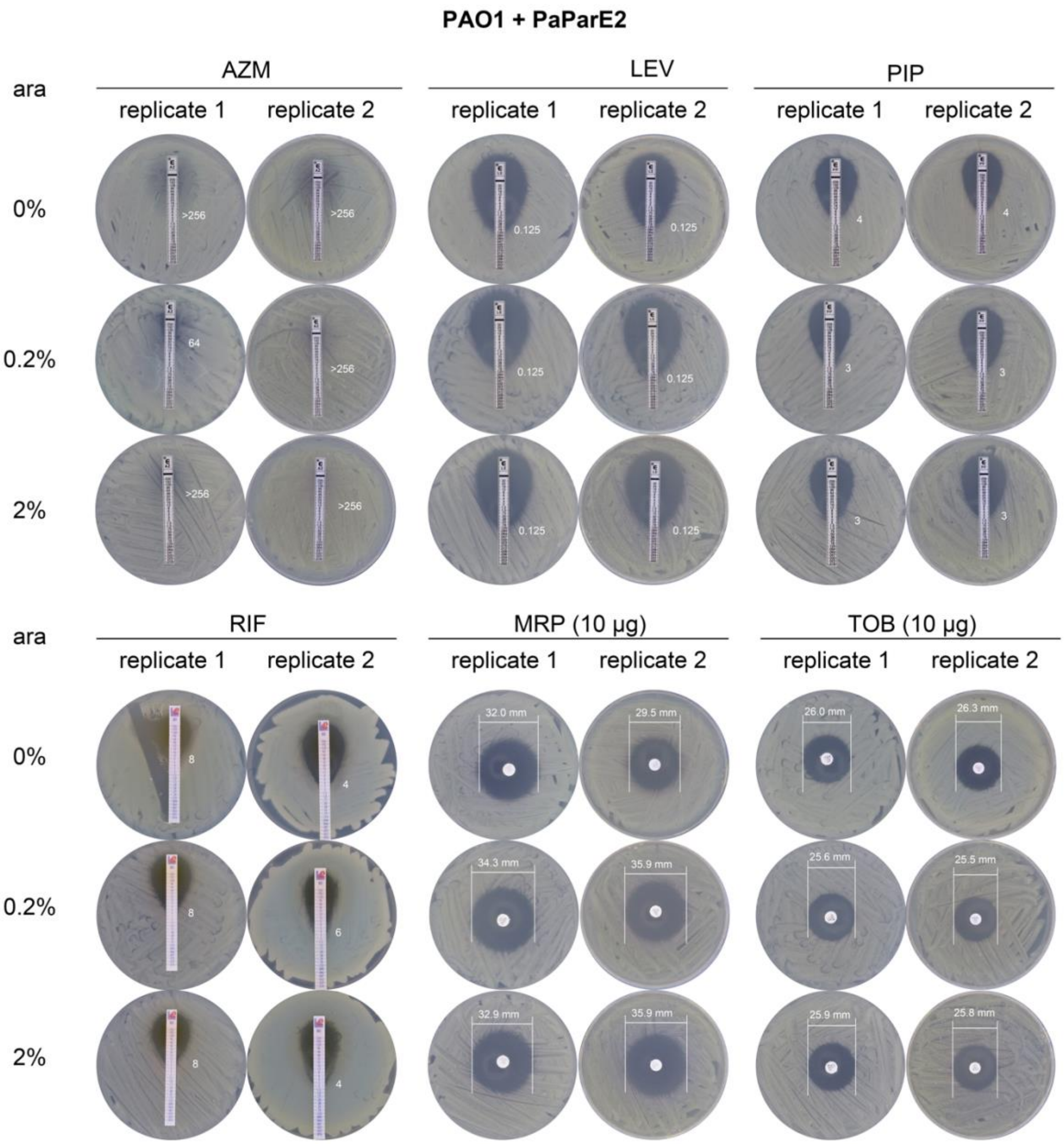

Figure S4. Impact of PaParE1 and PaParE2 toxin expression on *P. aeruginosa* PAO1 or PA14 cell antibiotic susceptibility. Cell cultures were swabbed onto test media plates after 8 hrs of PaParE expression at indicated arabinose (ara) induction strengths and then E-strips or discs of specific antibiotics were placed on the plates. Antibiotic susceptibilities were evaluated after 24-48 hrs growth by assessing the alteration in the size of the clear zone or by determining the limit of cleared growth indicating the MIC value as printed on strips. AZM: azithromycin; TOB: tobramycin; LEV: levofloxacin; PIP: piperacillin; MRP: meropenem.

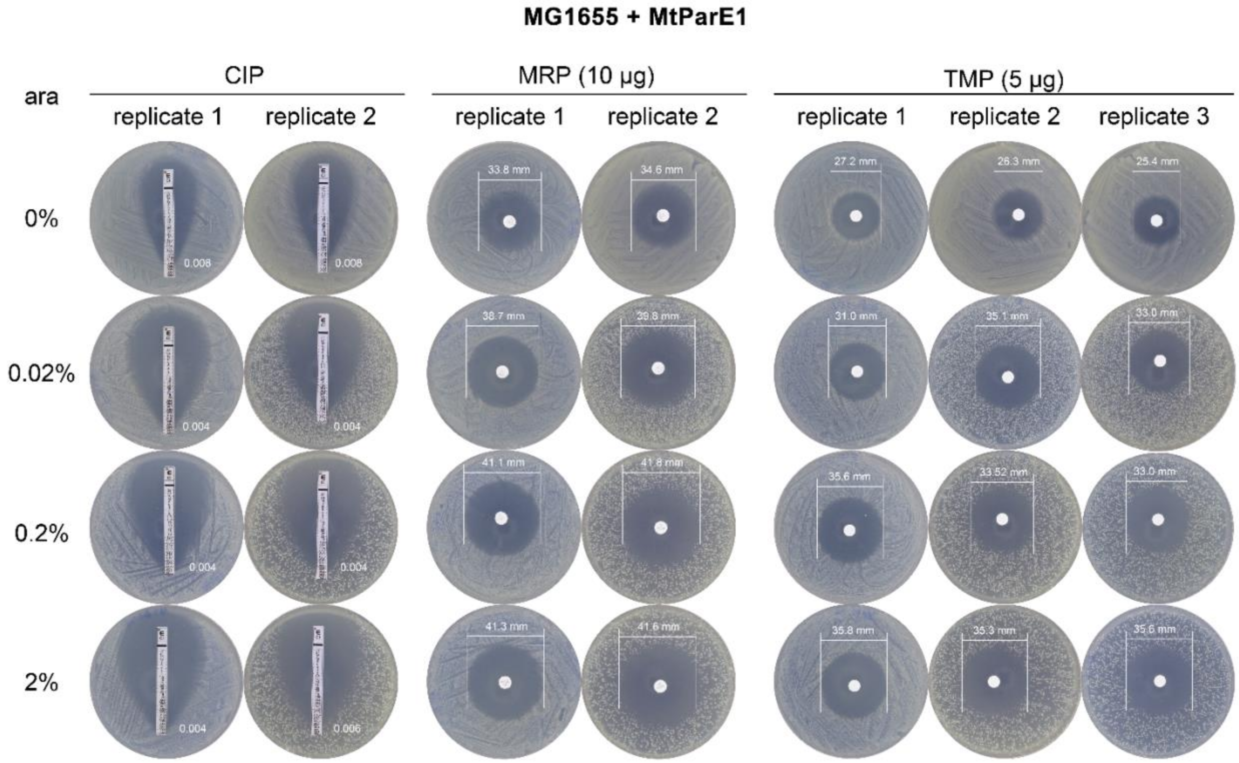

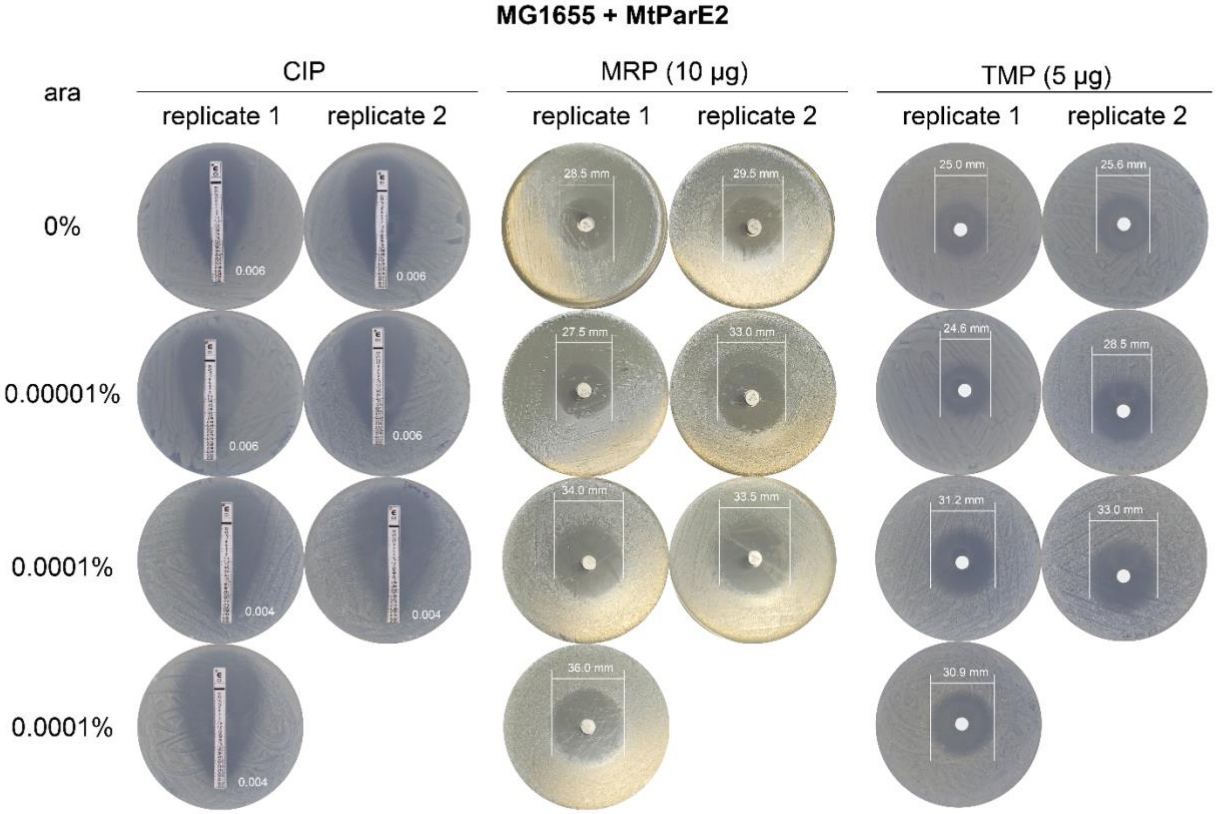

Figure S5. Impact of MtParE1 and MtParE2 toxin expression on *E. coli* MG1655 cell antibiotic

susceptibility. Cultures of *E. coli* cells expressing MtParE1 or MtParE2 were normalized for consistent cell density and swabbed onto LB agar plates following 8 hrs of MtParE expression at indicated arabinose (ara) concentrations, and discs or E-test strips of specified antibiotics were placed on the plates. Antibiotic susceptibilities were evaluated after 24 hrs growth by determining the diameter change of the inhibition (cleared) zones or by determining the limit of cleared growth indicating the MIC value as printed on strips. CIP: ciprofloxacin; MRP: meropenem; TMP: trimethoprim.

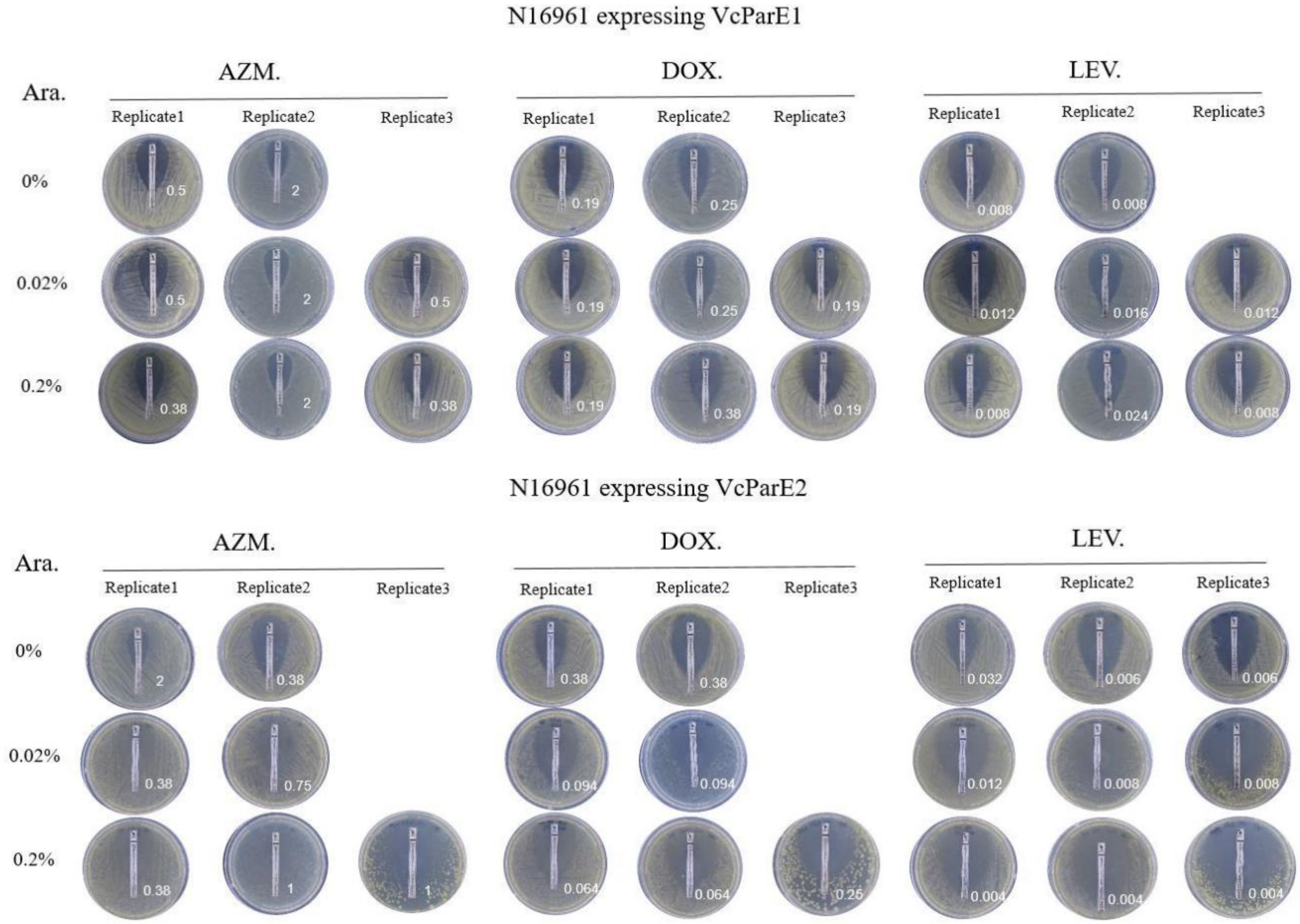

Figure S6. Impact of VcParE1 and VcParE2 toxin expression on *V. cholerae* N16961 cell antibiotic susceptibility. Cultures of *V. cholerae* cells expressing VcParE1 or VcParE2 were normalized for consistent cell density and swabbed onto LB agar plates following 8 hrs of expression at indicated arabinose (ara) concentrations, and E-test strips of specified antibiotics were placed on the plates. Antibiotic susceptibilities were evaluated after 8 hrs growth by determining the limit of cleared growth indicating the MIC value as printed on strips. AZM: azithromycin; DOX: doxycycline; LEV: levofloxacin.
